# Evaluation of the Cardiac Protection Conferred by Proanthocyanidins in Grape Seeds against Development of Ehrlich Solid Tumors in Mice

**DOI:** 10.1155/2020/3530296

**Published:** 2020-01-15

**Authors:** Maha Abdulrahman Aldubayan

**Affiliations:** Pharmacology and Toxicology Department, Faculty of Pharmacy, Qassim University, Buraydah, Saudi Arabia

## Abstract

Examination of the antineoplastic effects of a range of chemical compounds is often undertaken via the transplantable tumor model of Ehrlich solid tumor (EST), which is a simulation of breast cancer. The purpose of this study was to explore how cardiac toxicity, damage, oxidative stress, and changes in the expressions of TNF*α* and apoptotic P53 triggered by EST could be countered with grape seed proanthocyanidins (GSPE). To that end, 50 female mice were used, with arbitrary and equal distribution into five groups, namely, the control group (G1), GSPE group (G2), EST group (G3), GSPE + EST (G4; cotreatment consisted of mice that received GSPE treatment at the beginning of EST induction over a period of 14 days), and EST + GSPE (G5; posttreatment consisted of mice with EST that received GSPE treatment for 14 days following the 14 days since the induction of EST). By comparison with the control group, the EST group had significantly higher levels of serum lactate dehydrogenase (LDH), creatine phosphokinase (CPK), creatine kinase MB (CK-MB), myoglobin, cardiac TBARS, nitric oxide (NO), total thiol and hydrogen peroxide, cardiac damage, and expression of P53 and TNF*α*. On the other hand, the EST group had significantly lower levels of cardiac catalase and total antioxidant (TAC) than the control group. Furthermore, better improvement in cardiac toxicity, oxidative stress, damage, apoptosis, and TNF*α* expressions was displayed by the cotreated (GSPE + EST) group than by the posttreated (EST + GSPE) group. This led to the conclusion that GSPE conferred cardiac protective and antioxidant effects against EST. This finding calls for more investigation on the benefits of grape seeds as adjuvant agents to prevent and treat cardiac toxicity.

## 1. Introduction

Conditions underpinned by multiple factors (e.g., chronic heart failure, kidney failure, metabolic disorders, and cancer) lead to cardiac dysfunction [1, 2]. No other disease affecting humans is more severe and is associated with a greater mortality rate than cancer [3]. The onset of cancer is triggered owing to cell DNA mutations causing the formation of an extraneous tissue mass known as a tumor. At the same time, indirect activation of apoptosis is caused by numerous cancer treatments through chemical or physical damage to DNA [4]. Just like congestive heart conditions, cardiomyopathy triggered by cancer leads to cardiac dysfunction, which is associated with cardiac atrophy, metabolic remodelling, fibrosis, and cardiac ultrastructure alterations [5]. Among female individuals, the type of cancer with the highest prevalence is breast cancer, a disease with the second highest mortality rate [6]. Examination of the antineoplastic effects of a range of chemical compounds is often undertaken via the transplantable tumor model of Ehrlich solid tumor (EST), which is a simulation of breast cancer [6–8].

Chemotherapy and radiation are the basis of the majority of current cancer treatments, which work by triggering apoptosis to destroy cancer cells; however, this mechanism can also have an impact significantly on patient morbidity and mortality [9–11].

Many types of vegetables, seeds, and fruit contain the natural antioxidant proanthocyanidins, which comprises various phenolic compounds [12, 13]. Red grape seeds have been found to contain a number of polyphenolic antioxidants, with a proportion of proanthocyanidins of around 89%, including 6.6% dimers, 5% trimers, 2.9% tetramers, and 74.8% oligomers [14, 15]. The red grape seed extract known as grape seed proanthocyanidins extract (GSPE) presents various biological qualities, being effective against inflammation, apoptosis, proliferation, oxidants, and oxidative stress [14]. The purpose of this study was to investigate whether GSPE protected against the cardiac toxicity and oxidative stress caused by the Ehrlich solid tumor (EST).

## 2. Materials and Methods

### 2.1. Chemical and Reagent

  Grape seed proanthocyanidin (Gervital capsules with 96% purity of proanthocyanidin) were purchased from Arab company for pharmaceuticals and medicinal plants (MEPACO-MEDIFOOD, Anshas El Raml, Sharqeya, Egypt).
  Mice: the breeding unit of the Egyptian Organisation for Biological Products and Vaccines (Cairo, Egypt) supplied the female Swiss albino mice of adult age and with a weight of 22–25 g. The mice were fed a standard commercially available pellet diet and had unrestricted access to tap water. Approval for the experiment was obtained from the Local Ethics Committee and Animals Research at King Saud University, Riyadh, Saudi Arabia, and according to the guidelines for animal studies published by the Ethical Committee of Faculty of Science, Tanta University with the approval of the Institutional Animal Care and Use Committee (IACUC-SCI-TU-0041).

### 2.2. Experimental Design and Animal Groups

The 50 female mice were allowed to become accustomed to the experiment conditions for seven days before they were distributed arbitrarily in five groups that contained 10 animals each. Thus, the control group (G1) consisted of mice that were not treated in any way. The GSPE group (G2) consisted of mice that were administered daily by oral gavage 50 mg/kg GSPE over a period of 14 days [15]. The EST group (G3) consisted of mice that carried EST for a period of 14 days [16]. The GSPE + EST group (G4) consisted of mice that received GSPE treatment at the beginning of EST induction over a period of 14 days. The EST + GSPE group (G5) consisted of mice with EST that received GSPE treatment for 14 days following the 14 days since the induction of EST.

### 2.3. Ehrlich Ascites Carcinoma Cells and Tumor Induction

The Egyptian National Cancer Institute (NCI; Cairo University, Egypt) provided the mice that carried Ehrlich ascites carcinoma (EAC). To maintain the tumor line and evaluate EST, the mice used in the experiment were each injected intraperitoneally in the left thigh with 2.5 million cells in 0.2 ml volume per week. A bright line haemocytometer permitted quantification of the EAC cells before they were injected, and physiological sterile saline solution was used for dilution purposes.

### 2.4. Sample Collection

Upon completion of the experiment, all mice of each group were anaesthetized with sodium pentobarbital, decapitated, and then dissected. Blood was sampled from the inferior vena cava of each mouse and stored in nonheparinised glass tubes to estimate cardiac biomarkers. On the other hand; hearts were removed from mice in different groups and used for measurement the cardiac oxidative stress, histopathology, and immunohistochemistry investigation. Half of isolated hearts were homogenized and used for cardiac oxidative stress, while the rest were washed in 0.9% saline solution and fixed in 10% neutral buffered formalin and used for histopathology and immunohistochemistry investigation.

### 2.5. Measurement of Cardiac Biomarkers

The activity of serum lactate dehydrogenase (LDH) was determined using an assay kit (Vitro Scient, Cairo, Egypt) according to the method of Whitaker [17]; meanwhile, serum creatine phosphokinase (CPK) was determined using an akinetic technique with kits (Vitro Scient, Cairo, Egypt) based on the method of Salama et al. [18]. Serum levels of creatine kinase MB (CK-MB) were determined using an assay kit (Bioassay Systems, Hayward, CA, USA) according to the method of Bishop et al. [19]. The myoglobin concentration in serum was assayed using a kit (Reactivos Spinreact, Girona, Spain) according to the method of Cummins et al. [20].

### 2.6. Measurement of the Biomarkers of Cardiac Oxidative Stress

Half of the isolated hearts were weighed and Potter–Elvehjem-type homogenizer was used for homogenization by adding potassium phosphate buffer (pH 7.4) and ice-cold 1.15% KCl-0.01 mol/L sodium to the heart tissues. The supernatant was obtained by centrifugation of homogenate at 10,000×g for 20 min. at 4°C and the resultant supernatant was used for analysis.

Thiobarbituric acid-reactive substances (TBARS) were measured in cardaic homogenate using the method of Oyouni et al. [21]. The enzyme catalase (CAT; EC 1.11.1.6) converts H_2_O_2_ into water. The CAT activity was measured spectrophotometrically at 240 nm by calculating the rate of degradation of H_2_O_2_, the substrate of the enzyme [22]. Hydrogen peroxide (H_2_O_2_) was measured in cardiac homogenate using the method of Saggu et al. [23]. Total thiol was measured using DTNB reagent according to Sedlak and Lindsay [24]. Nitric oxide (NO) was measured in cardiac homogenate using the method of Beltagy et al. [25]. The total antioxidant capacity (TAC) was measured using the ferric reducing antioxidant power [26].

### 2.7. Histopathological Investigation

After mice dissection, the hearts were removed from mice in different groups, half of isolated hearts were fixed in 10% buffer neutral formalin, dehydrated in an increasing series of ethyl alcohol, cleared in two changes of xylene, and embedded in molten paraffin at a temperature of 50–58°C. A rotary microtome was used to cut 7 *μ*m sections, which were placed on clean slides and subjected to staining with Ehrlich's haematoxylin [27].

### 2.8. Detection of P53 and TNF*α* Expressions

For the purposes of detection of the expression of apoptotic p53 proteins and tumor necrosis factor-*α* (TNF*α*) in the heart sections, the method of avidin Biotin Complex (ABC) (Elite–ABC, Vector Laboratories, CA, USA) was applied to P53 (dilution 1 : 200 DAKO Japan Co., Ltd., Tokyo, Japan) and TNF*α* (rabbit polyclonal IgG, 100 *μ*g/ml, 1 : 50 dilution, cat. no. sc-130220; Santa Cruz Biotechnology, Inc., Dallas, TX, USA) [12, 28].

### 2.9. Statistical Analysis

The statistical software program Prism (GraphPad.Prism.v5.01) was employed to conduct statistical analysis. Results were analyzed using one-way analysis of variance (ANOVA) followed by the Least Significant Difference (LSD) tests to compare between different groups. Data were presented as the mean ± SEM. *P* values less than 0.01 were considered significant. All statistical analyses were performed using SPSS statistical version 16 software package (SPSS® Inc., USA).

## 3. Results

### 3.1. Toxicity

Throughout the research period, no mouse in the control group or GSPE group died and the mice in the experimental groups did not exhibit any secondary effects due to GSPE administration.

### 3.2. Effect of GSPE on Cardiac Biochemical Markers

**Figure 1:**
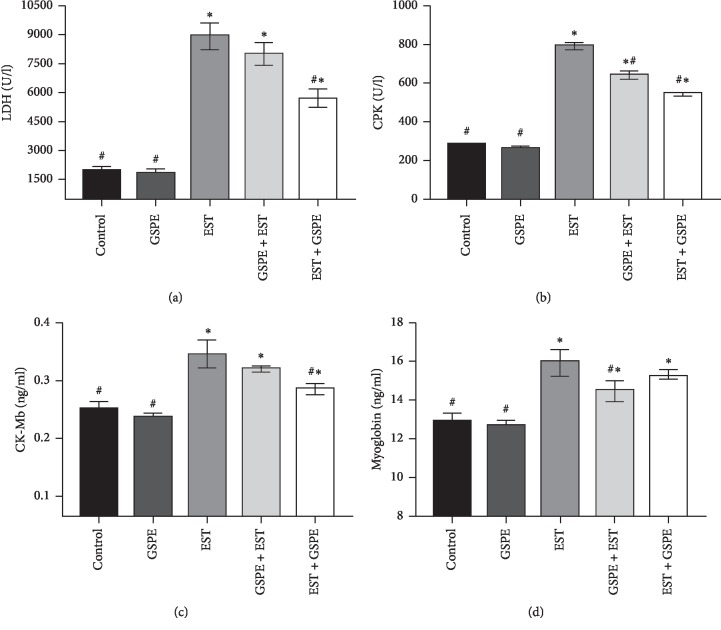
Changes in cardiac function parameters (serum LDH, CPK, CK-MB, and myoglobin) levels in different experimental groups; expression of values takes the form of means ± SEM, with 10 subjects in every treatment group; ^#^*P* < 0.01 Significant difference from EST, ^*∗*^*P* < 0.01 Significant different from control.

As shown in Figure 1, by comparison with the control group and the GSPE group, the EST group displayed significantly (*P* < 0.01) higher levels of LDH, CPK, CK-MB, and myoglobin, but treatment of EST with GSPE significantly reduced those levels. By contrast, the EST + GSPE group had significantly (*P* < 0.01) lower levels of LDH, CPK, and CK-MB than the GSPE + EST group.

### 3.3. Effect of GSPE on Oxidative Stress

**Figure 2:**
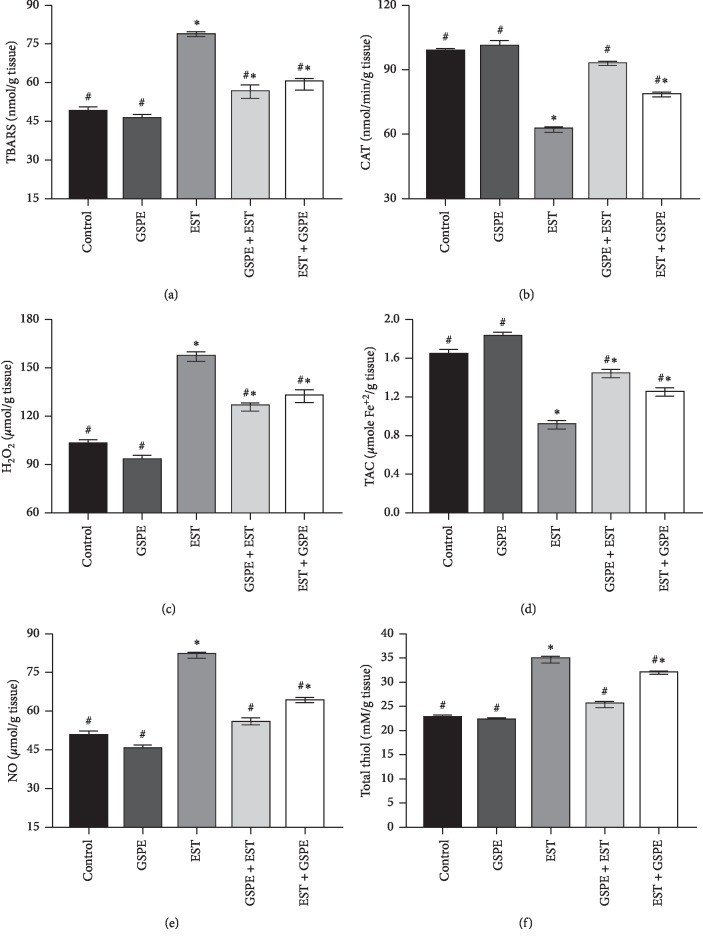
Alterations in the oxidative stress parameters (TBARS, total thiol, hydrogen peroxide, nitric oxide, catalase, and TAC) in cardiac tissues derived from the different groups. Expression of values takes the form of means ± SEM, with 10 subjects in every treatment group; ^#^*P* < 0.01 Significant difference from EST, ^*∗*^*P* < 0.01 Significant different from control.

As can be seen in Figure 2, by comparison with the control group and the GSPE group, the EST group had significantly (*P* < 0.01) higher levels of TBARS, total thiol, hydrogen peroxide, and nitric oxide, but significantly lower levels of cardiac catalase and TAC. However, GSPE treatment helped to return all the abovementioned parameters to normal levels. Furthermore, by contrast to the EST + GSPE group, the GSPE + EST group displayed significantly lower levels of cardiac TBARS, total thiol, hydrogen peroxide, and nitric oxide levels, but significantly higher levels of cardiac catalase and TAC.

### 3.4. Effect of GSPE on Heart Histopathology

**Figure 3:**
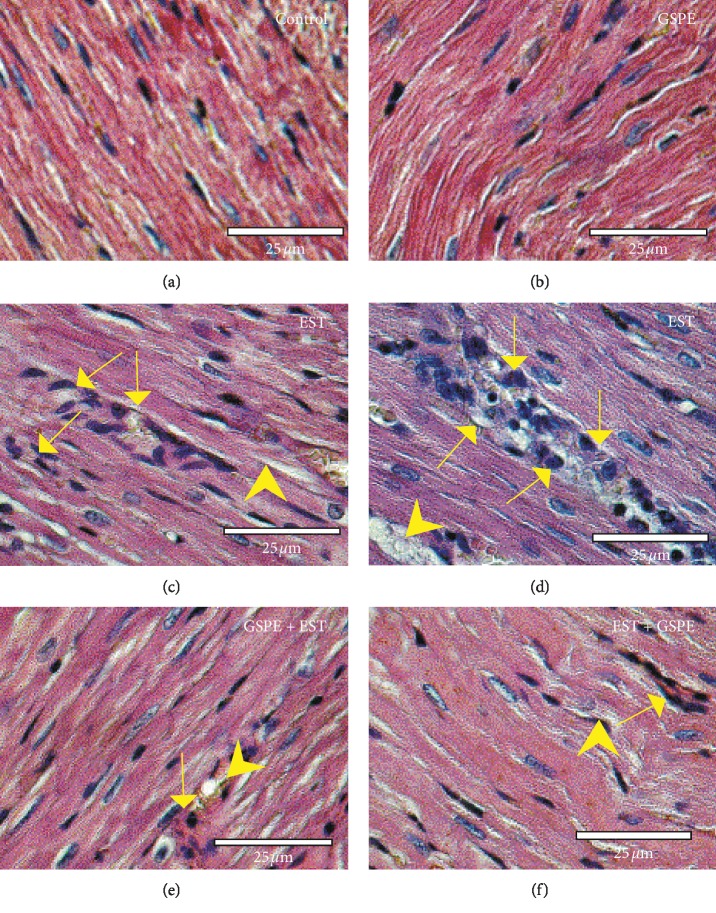
Photomicrographs of haematoxylin- and eosin-stained heart sections from mice in different groups. (a, b) Normal myofibrillar structure with striations shown by heart sections from mice in the control and GSPE groups. (c, d) Significant myocardial hypertrophy (arrow heads), nuclear pyknosis, focal haemorrhage, marked cytoplasmic vacuoles, and leukocyte infiltration (arrows) exhibited by heart sections from mice in the EST group. (e) Mild tissue damage with mild myocardial hypertrophy (arrow heads) and nuclear pyknosis (arrows) exhibited by heart sections from mice in the GSPE + EST group. (f) Mild myocardial hypertrophy (arrow heads), mild cytoplasmic vacuoles, and mild leukocyte infiltration (arrows) displayed by the heart sections from mice in the EST + GSPE group.

As illustrated in Figures 3(a) & 3(b), a regular myofibrillar structure with striations was exhibited by heart sections from mice in the control group and the GSPE group, whereas Figures 3(c) & 3(d)) show that there were significant myocardial hypertrophy, nuclear pyknosis, focal haemorrhage, marked cytoplasmic vacuoles, and leukocyte infiltration in the heart sections from mice in the EST group. By contrast, Figure 3(e) reveals that there was mild tissue damage with mild myocardial hypertrophy and nuclear pyknosis in heart sections from mice in the GSPE + EST group, while Figure 3(f) shows that there was mild myocardial hypertrophy, mild cytoplasmic vacuoles, and mild leukocyte infiltration in the heart sections from mice in the EST + GSPE group.

### 3.5. Changes in P53 Expression in Heart

**Figure 4:**
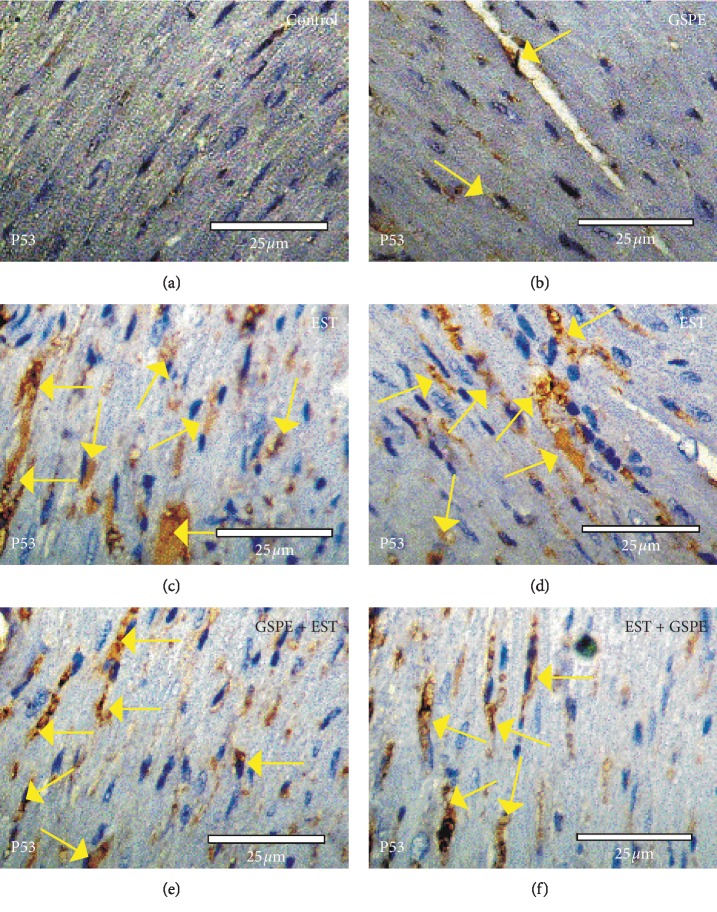
Photomicrographs of heart sections in the different experimental groups stained with P53 expression. (a, b) Heart sections from mice in the control group and GSPE group exhibiting slight positive P53 reactions (arrows). (c, d) Heart sections from mice in the EST group displaying strong positive reactions (arrows) for P53 expression. (e, f) Heart sections from mice in the GSPE + EST and EST + GSPE groups exhibiting moderate-to-mild positive reactions (arrows) for P53 expression.

There was a slight positive reaction for P53 expression in the heart sections from mice in the control group and the GSPE group (Figures 4(a) & 4(b)), while the heart sections from mice in the EST group exhibited strong positive reactions for P53 expression (Figures 4(c) & 4(d)). By contrast, mild-to-moderate positive reactions for P53 expression were observed in the heart sections from mice in the GSPE + EST group and EST + GSPE group (Figures 4(e) & 4(f)).

### 3.6. Changes in TNF*α* Expression in Heart

**Figure 5:**
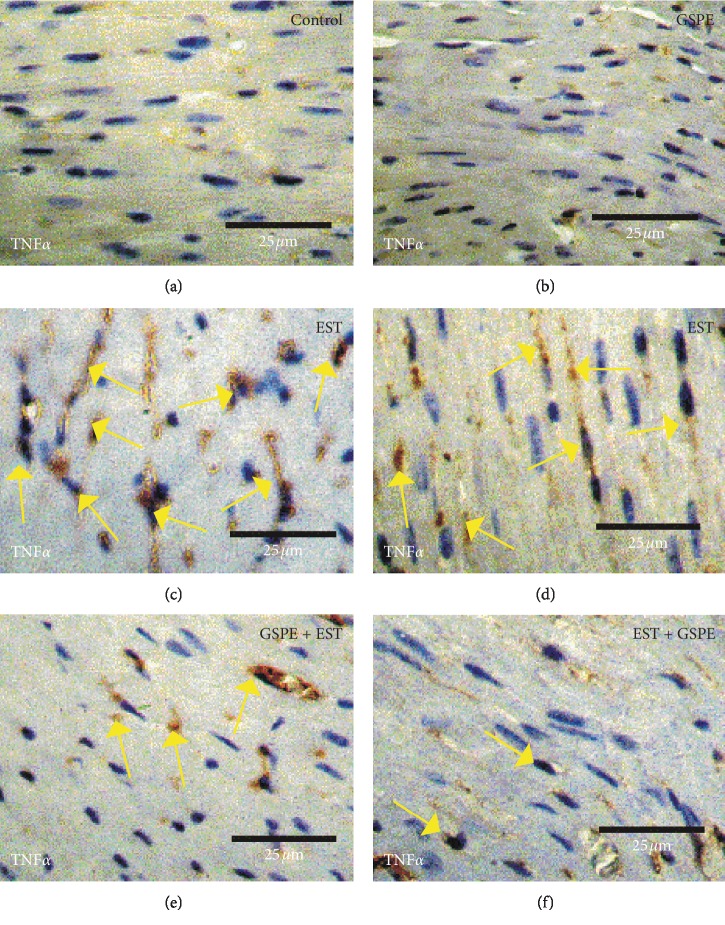
Photomicrographs of heart sections in the different experimental groups stained with TNF*α*. (a, b) Heart sections from mice in the control group and GSPE group exhibiting slight positive TNF*α* reactions (arrows). (c, d) Heart sections from mice in the EST group displaying moderate positive reactions (arrows) for TNF*α*. (e, f) Heart sections from mice in the GSPE + EST and EST + GSPE groups exhibited mild positive reactions (arrows) for TNF*α* expression.

Negative or slight positive reaction for TNF*α* expression was observed in the heart sections from mice in the control group and the GSPE group (Figures 5(a) & 5(b)), whereas the heart sections from mice in the EST group displayed moderate positive reactions for TNF*α* expression (Figures 5(c) & 5(d)). By contrast, mild-to-moderate positive reactions for TNF*α* expression were observed in heart sections from mice in the GSPE + EST group and the EST + GSPE group (Figure 5(e) & 5(f)).

## 4. Discussion

Resembling human breast cancer, Ehrlich carcinoma represents an undifferentiated carcinoma that starts out as hyperdiploid, has a high capacity for transplantation, does not regress, proliferates quickly, has a brief life-span, is fully malignant, and lacks a tumour-specific transplantation antigen [29]. The purpose of this study was to investigate the extent to which GSPE had a protective effect against cardiac toxicity, oxidative stress, damage, apoptosis, and TNF*α* alterations caused by EST in mice. This is the first publication describing a method to test how breast cancer affects cardiac structure and function using a novel mice model. The results obtained suggested that cardiac function, as well as the function of other important organs, was indeed affected by tumor formation. More specifically, by comparison with the control group, the EST group exhibited significantly higher levels of LDH, CK, CK-Mb, and myoglobin in the serum (*P* < 0.01), providing evidence that cardiac dysfunction in mice was caused by EST.

Tumour onset and metabolism are underpinned by LDH. In the present study, hepatotoxicity, cardiac diseases, and/or rapid tumor cell metabolism could all have been the cause of the high levels of LDH exhibited by mice carrying EST. The results obtained were consistent with those reported by Noureldeen et al. [30] and Qusti et al. [31], who found that Ehrlich ascites carcinoma was associated with high levels of cardiac enzymes. Likewise, cardiomyopathy was attributed to subcutaneous Ehrlich ascites carcinoma in the study by Aldubayan et al. [8]. Furthermore, leakage of CK-MB occurs when the myocardium is severely injured, which in turn is the outcome of contractile apparatus breakdown and greater sarcoplasmic permeability.

Grapes are rich in polyphenol compounds, such as flavonoids, phenolic acids, and resveratrol. The cardiovascular benefits of inclusion of these compounds in the diet enjoy significant epidemiological support [32, 33]. This study observed that GSPE treatment of EST led to a significant reduction in the high levels of cardiac enzymes, but the GSPE + EST group had significantly lower levels of LDH, CK, and CK-MB than the EST + GSPE group. Thus, it was concluded that cardiotoxicity caused by EST could be attenuated with GSPE. Furthermore, grape polyphenols were shown by a large number of *in vitro*, animal, and human studies to have a positive effect on prevalent cardiovascular risk factors [34]. The findings of this study were consistent with those reported by Tousson et al. [15], who observed that cardiac toxicity caused by boldenone undecylenate was attenuated by GSPE by suppressing NADPH oxidase and downregulating NOX2 and NOX4 expression. Moreover, based on rodent models, GSPE has been demonstrated by Karthikeyan et al. [35] to confer effective cardiac protection by ameliorating myocardial damage caused by isoproterenol.

Carcinogenesis begins, develops, and advances with the involvement of oxidative mechanisms [36–38]. Unlike normal cells, the levels of reactive oxygen species (ROS) are higher in cancer cells, leading to elevated oxidative stress that damages the components of the cells and eventually causes the cells to die [3, 8]. The findings of the present study indicated that, by comparison with the control group and the GSPE group, the EST group had significantly higher levels of cardiac TBARS, total thiol, hydrogen peroxide, and nitric oxide, but significantly lower levels of cardiac catalase and TAC (*P* < 0.01). Cancer cells trigger free radicals to be produced in excess, damaging lipids, and potentially triggering lipid peroxidation, which may be the reason for the notable rise in the levels of cardiac MDA exhibited by the EST group in this study [39].

The findings of this study are also consistent with those of Noureldeen et al. [30], who injected EAC cells in mice and observed that cardiac tissues exhibited a marked decrease in TAC and marked increase in the levels of malondialdehyde. Furthermore, there was good agreement between the findings of this study and those of Nisari et al. [40], who revealed that the levels of MDA in the liver, kidney, and testis were heightened by EST formation. Moreover, Noureldeen et al. [30] found that, by comparison with healthy mice, mice carrying tumour had significantly higher levels of cardiac catalase. These results were corroborated by the present study, which observed that animals carrying tumour had lower TAC and higher levels of malondialdehyde, as TAC is indicative of the overall levels of oxidative stress in the body [41, 42].

GSPE treatment of EST reversed the increase in cardiac TBARS, total thiol, hydrogen peroxide, and nitric oxide levels and the reduction in catalase and TAC. Meanwhile, by comparison with the EST + GSPE group, the GSPE + EST group exhibited significantly lower levels of cardiac TBARS, total thiol, hydrogen peroxide, and nitric oxide and significantly higher levels of cardiac catalase and TAC. Fitzpatrick et al. [43] reported that vascular endothelium produced more nitric oxide *in vitro* due to the effect of oligomeric proanthocyanidin extract from other sources (e.g., Pycnogenol from pine bark). Moreover, *in vitro* research has reported that *ex vivo* LDL oxidation was reduced by flavonoids and resveratrol from grapes [44]. Additionally, the results of this study were congruous with those of Kandemir et al. [45], who found that grape seed extract acted as a protective antioxidant agent against rabbit liver toxicity caused by cisplatin.

The outcomes of cardiac tissue histopathology and immunohistochemistry backed up the abovementioned results; significant myocardial hypertrophy, apoptosis-indicative nuclear myocardial pyknosis, focal haemorrhage, marked cytoplasmic vacuoles, and leukocyte infiltration reflected cardiac damage in the heart section of mice with EST, implying that cardiac fibrosis was caused by subcutaneous EST owing to excessive myofibroblast production. This finding was consistent with Noureldeen et al. [30], who observed that cardiac damage was caused by EAC, with notable nuclear myocardial pyknosis and cellular infiltrations.

Cardiac damage was ameliorated by GSPE treatment of EST, while better heart tissue improvement was achieved by GSPE + EST compared with EST + GSPE. Thus, it was deduced that cardiotoxicity and cardiac injury caused by EST were prevented by GSPE. Comprising a mix of bioflavonoids with biological activity (e.g., oligomeric proanthocyanidins), GSPE can have protective effect against hypertrophy and cellular infiltrations of cardiac muscle fibre cells caused by EST by diminishing the levels of free radicals and lipid peroxidation. From this perspective, the study concurred not only with Razmaraii et al. [46], who found that GSPE protected against cardiotoxicity caused by doxorubicin in Wistar rats, but also with Lian et al. [47], who observed that cardiotoxicity, lipid peroxidation, oxidative stress, and cardiac damage caused by cisplatin were alleviated by GSPE.

There is an inextricable link between tumor development and heightened proliferation, so a compound suppressing proliferation and triggering apoptosis is likely to hinder tumor development. The apoptosis-inducing effect of EST was confirmed in this study by the marked-up regulation in the expression of p53 and TNF*α* in heart tissue, while GSPE treatment had a modulating effect on this change. Through modulation of regulatory genes of cell cycle/apoptosis (e.g., bcl-2, p53, and c-myc), GSPE has a protective effect against cytotoxicity caused by chemotherapy medication in human hepatic cells [48]. In this study, cardiotoxicity and oxidative stress were alleviated and expression of p53 and TNF*α* was reduced by GSPE treatment of EST. Our results agree with Eldaim et al. [7] who reported that EST induced alterations in kidney P53, PCNA, and KI67 expressions and grape seeds proanthocyanidin extract ameliorates this alteration in mice kidney and also with Ali et al. [49] who reported that grape seeds and skin have antioxidant and hepatoprotective activities against Ehrlich solid tumor induced oxidative stress in mice. GSPE, may be due to its antioxidant activity, appears to reduce the inflammatory processes which might partly explain the mechanism(s) for the amelioration of other chronic inflammatory conditions such as inflammatory bowel disease, cancer, and diabetes. The scavenging process was thus improved, enabling the removal of ROS or free radicals produced as the tumor progressed. Our findings might help to better understand the mechanism of cardiac injury during EST and provide novel targets for evaluating the effects of GSPE therapy. This finding calls for more investigation on the benefits of grape seeds as adjuvant agents to prevent and treat cardiac toxicity.

## Data Availability

The data used to support the findings of this study are available from the corresponding author upon request.
